# Effects of siRNA-mediated HIF-1α gene silencing on angiogenesis in osteosarcoma

**DOI:** 10.12669/pjms.332.12587

**Published:** 2017

**Authors:** Xu-dong Zhang, Qiang Wu, Shu-hua Yang

**Affiliations:** 1 Dr. Xu-dong Zhang, Department of Orthopedics, Union Hospital, Tongji Medical College, Huazhong University of Science and Technology, Wuhan, China; 2 Prof. Qiang Wu, Department of Orthopedics, Union Hospital, Tongji Medical College, Huazhong University of Science and Technology, Wuhan, China; 3 Prof. Shu-hua Yang, Department of Orthopedics, Union Hospital, Tongji Medical College, Huazhong University of Science and Technology, Wuhan, China

**Keywords:** Osteosarcoma, Angiogenesis, Vascular endothelial growth factor, CD34, Hypoxia-inducible factor-1α, Gene silencing, Small interference RNA

## Abstract

**Objective:**

To explore angiogenesis in osteosarcoma under the condition of hypoxia-inducible factor (HIF)-1α gene silenced by small interference RNA (siRNA).

**Methods:**

The SaOS-2 osteosarcoma cells, transfected with the recombinant plasmid pSilencer2.1-HIF-1α or pSilencer2.1-SCR, were classified as HIF-1α/siRNA group or SCR/siRNA group, respectively. In which, vascular endothelial growth factor (VEGF) immunohistochemistry were performed. HIF-1α and VEGF protein contents were detected by western blot. Gene expressions of HIF-1α and VEGF were quantified by qPCR. Then the transfected SaOS-2 cells were inoculated in nude mice and transplantation tumor were checked via HE staining, VEGF and CD34 immunohistochemistry, and calculation of microvascular density (MVD).

**Results:**

In vitro, VEGF immunohistochemistry stains, HIF-1α and VEGF protein contents, and the relative expressions of HIF-1α mRNA and VEGF mRNA in HIF-1α/siRNA group were obviously reduced. In vivo, morphological observation illustrated that heteromorphism were not obvious in the cells of HIF-1α/siRNA group and vascular systems were sparse in its transplantation tumor tissue, and immunohistochemistry revealed that both VEGF and CD34 stains were significantly decreased in HIF-1α/siRNA group, and MVD in HIF-1α/siRNA group (7.3±1.1) were obviously less than that in SCR/siRNA group (17.2±3.2) (*P*<0.05).

**Conclusion:**

Angiogenesis in osteosarcoma can be inhibited by siRNA-mediated HIF-1α gene silencing, which is expected to provide a novel and attractive target of therapeutic strategies of osteosarcoma.

## INTRODUCTION

Osteosarcoma, like other neoplasms, with expansion beyond a certain tumor burden necessitates angiogenesis which provides additional oxygen and nutrients.^1^ Conceivably, osteosarcoma characteristically contains some regions of oxygen deprivation. Hypoxia activates and mediates the heterodimeric transcription factor termed hypoxia-inducible factor-1 (HIF-1).^2^ HIF-1 is composed of α and β subunits, and HIF-1α triggers the biological behavior of tumor cells under hypoxic conditions, including proliferation, invasion, metastasis, enhanced resistance to chemotherapy, recurrence, and poor clinical prognosis.^3^-^5^

Fire *et al* had firstly found and proposed RNA interference (RNAi) that dsRNA homologous endogenous gene expression was silenced after injecting the dsRNA into Caenorhabditis elegans.^6^ Utilized as an effortless and effective procedure instead of gene knockout, RNAi provides an approach available for gene therapy.^7^

Angiogenesis, new blood vessels developed from preexisting vessels within a living organism, is crucial during tumorigenesis and tumor progression.^8^ This process is exceedingly regulated by vascular endothelial growth factor (VEGF), the principal proangiogenic factor which gene expression is transcriptionally activated by HIF-1α.^9^-^11^ Whereas under the HIF-1α gene silencing condition, angiogenesis in osteosarcoma remains elusive. In this study we explored the influence of HIF-1α gene silencing on angiogenesis in osteosarcoma.

## METHODS

The SaOS-2 human osteosarcoma cell line was obtained from China Center for Type Culture Collection, and cultured in DMEM medium containing 10% fetal bovine serum plus 150µm/L CoCl_2_ at 37°C in the ambient air with 1% O_2_, 5% CO_2_, and 94% N_2_. The eukaryotic expression vector pSilencer™ Neo U_6_ 2.1 was purchased from Ambion (USA). Liposome Lipofectamin™ was from Invitrogen (USA). G418, RT-PCR kit, HIF-1α antibody, VEGF antibody, CD34 antibody, Gapdh antibody, and other related reagents were procured from Beijing Zhongshan Biotechnology Company (China). Balb/C mice were supplied by the Experimental Animal Center of Tongji Medical College (China). All procedures involved in mice were performed under the supervision and approval of the animal ethics committee.

### Plasmid construction and transfection

According to the siRNA targeting HIF-1α gene sites: AAAGAGGTGGATATGTCTGGG, which were found in gene bank and designed by ambion RNAi design software (USA), two synthetic complementary siRNA oligonucleotides were synthesized and annealed by Sangon Biotech (China), then connected with linearized plasmid pSilencer™ Neo U_6_ 2.1, creating recombinant plasmid called pSilencer2.1-HIF-1α. In the same way, pSilencer2.1-SCR was constructed with scramble siRNA which gene sites: GACATCAGTCGACATCAGA. The SaOS-2 cells were, respectively, stably transfected with pSilencer2.1-HIF-1α or pSilencer2.1-SCR mediated by Lipofectemin™, according to the manufacturer’s instructions. The resistant clone cells were screened in G418 selective culture-medium and harvested until three weeks. The SaOS-2 cells transfected with pSilencer2.1-HIF-1α or pSilencer2.1-SCR were classified as HIF-1α/siRNA group or SCR/siRNA group.

### VEGF immunohistochemistry

The cells of each group and the tumor tissue specimens of the experimental nude mice were fixed in formalin. The tissue specimens were paraffin-embedded and serially sectioned into 4 µm slices. Then paraffin sections were deparaffinized in xylene, rehydrated in grade alcohols and rinsed in double distilled water for immunohistochemical staining. The VEGF protein in all specimens was detected by streptavidin-biotin-peroxidase method according to the kit instructions. Negative control was set up by substituting the primary antibody with PBS solution. The VEGF-positive cells were stained brown and stains were predominantly found in the cytoplasm.

### Western blot

The cultured cells were harvested, lysed and centrifugated. The 20 μg supernatant protein were quantified by the Lowry method, mixed with SDS-PAGE sample buffer and mercaptoethanol, incubated at 100°C for 5 minutes, separated by 15% SDS-PAGE and transferred to PVDF membrane. The membrane blocked in 5% fat-free milk was incubated with 1:250 rabbit antihuman HIF-1α antibody, VEGF antibody, and Gapdh antibody at 20°C for 2 hours and then with 1:3,000 HRP conjugated secondary antibody at 20°C for 1 hours. After membrane was thrice washed in TBST for interval of 10 minutes, bands of HIF-1α, VEGF, or Gapdh protein were visualized with ECL chromogenic substrate. The relative levels of these proteins to control Gapdh were probed with image J software.

### Quantitative real-time PCR analysis

HIF-1α and VEGF mRNA expressions were quantified by qPCR using RT-PCR kits, according to the manufacturer’s instruction. The primers designed per PRIMER 5.0 software were synthesized by Sangon Biotech (for HIF-1α: forward primer 5’-TCTGGGTTGAAACTCAAGCAACTG-3’ and reverse primer 5’-CAACCGGTTTAAGGACACATTCTG-3’; for VEGF: forward primer 5’-AGGAGGAGGGCAGAATCATCA-3’ and reverse primer 5’-CTCGATTGGATGGCAGTAGCT-3’; for Gapdh: forward primer 5’-TTCACCACCATGGAGAAGGC-3’ and reverse primer 5’-GGCATGGACTGTGGTCATGA-3’). qPCR analyses were performed with cDNA reaction as a template, employing the StepOnePlus Real-Time PCR System (Applied Biosystems, USA). The ΔΔCT methods using Gapdh as a reference were utilized to compute the relative expression of HIF-1α or VEGF. The procedures were entirely performed thrice using independent samples.

### Transplantation tumor experiment in nude mice

The HIF-1α/siRNA or SCR/siRNA group cells in each well were, respectively, 1×10^7^ at the logarithmic growth phase, washed twice using PBS, and adjusted to 0.3 ml cell suspensions. The cell suspensions were, respectively, subcutaneously inoculated in two groups (*n*=12 each) of 4-week-old Balb/C female mice. The origination of oncogenesis and the capacity of tumor were continuously observed and recorded for four weeks. Once mice were sacrificed, tumor tissues were checked utilizing routine hematoxylin and eosin staining and immunohistochemical staining of VEGF or CD34. The tumor microvascular density (MVD) was calculated by tumor capillaries and small blood vessels which were marked with CD34. According to the judgment standard of Weidner, brown single endothelial cells or cell clusters were considered as a vessel, but the thick muscle layers and the vessels whose lumen area are greater than 8 times of erythrocyte diameter were not counted.^12^ Briefly, the whole section was detected in low power lens (10×10) and the three horizons of the most intensive area (microvascular angiogenic hotspots) were detected. At high magnification visual range (10×40), all stained microvasculatures were counted. The average count of the results at three fields of the vision was considered as microvascular count of the slice.

### Statistical analysis

Utilizing SPSS15.0 software package, statistical significance was determined by the *P* value of one-way ANOVA less than 0.05.

## RESULTS

**Fig.1:**
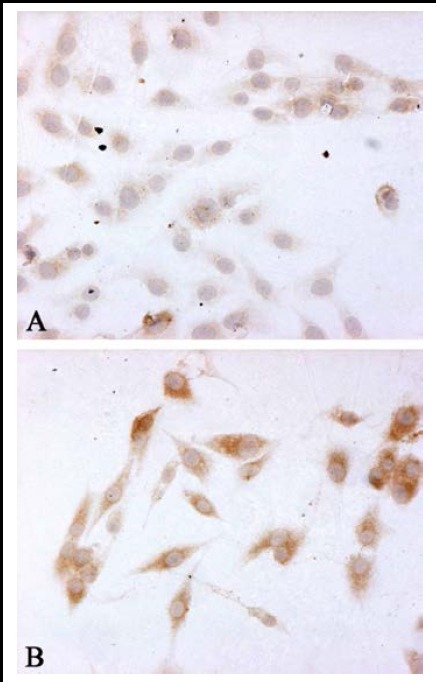
VEGF immunohistochemistry in vitro (original magnification: ×200). A: HIF-1α/siRNA group; B: SCR/siRNA group.

The VEGF proteins in two groups were observed using immunohistochemical staining. The proteins were localized in cytoplasm and displayed brown particles. The results showed that brown particles were significantly reduced in the cells of HIF-1α/siRNA group, but robustly expressed in SCR/siRNA group. (Fig.1)

**Fig.2:**
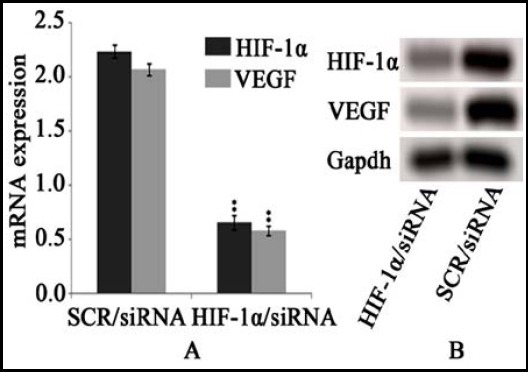
Analyses of HIF-1α and VEGF in vitro A: HIF-1α and VEGF mRNA expression; B: HIF-1α and VEGF protein contents.

According to Western blot, HIF-1α and VEGF protein contents in HIF-1α/siRNA group were simultaneously diminished compared to SCR/siRNA group (*P*<0.05). qPCR analysis demonstrated the relative expression of HIF-1α and VEGF in HIF-1α/siRNA group were statistically significantly reduced compared to SCR/siRNA group (*P*<0.01). (Fig.2)

**Fig.3:**
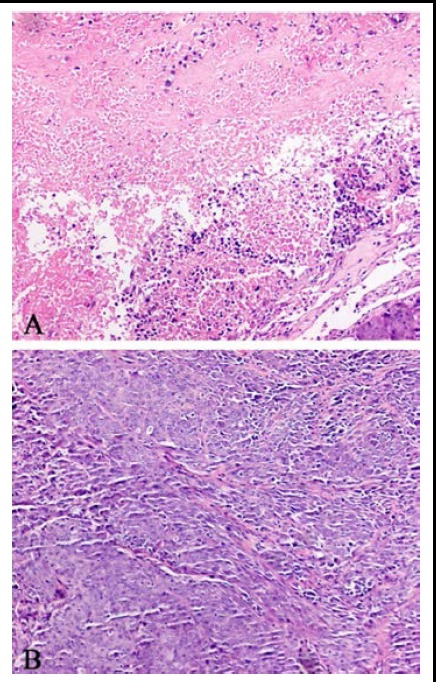
Morphological observation in vivo by HE staining (original magnification:×100). A: HIF-1α/siRNA group; B: SCR/siRNA group.

Heteromorphism was not obvious in the transplantation tumor cells of HIF-1α/siRNA group and vascular systems were sparse in its tissues, but in SCR/siRNA group heteromorphism was obvious and vascular systems were increased. (Fig.3)

**Fig.4:**
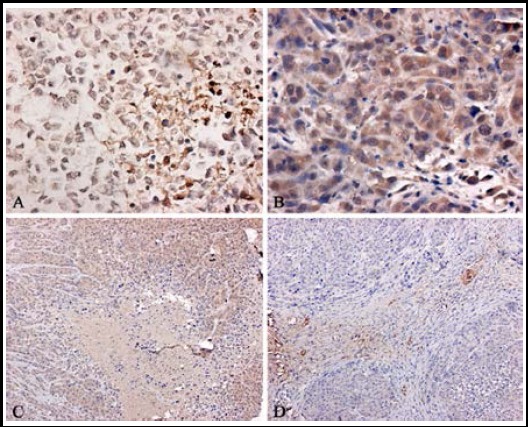
Immunohistochemistry in vivo (original magnification:×400) A: VEGF in HIF-1α/siRNA group; B: VEGF in SCR/siRNA group; C: CD34 in HIF-1α/siRNA group; D: CD34 in SCR/siRNA group.

The brown particles of VEGF significantly decreased in HIF-1α/siRNA group while it were obviously displayed in SCR/siRNA group. CD34 immunohistochemistry showed that the brown yellow stains were localized to the endothelium and CD34 stains were markedly reduced in HIF-1α/siRNA group compared to SCR/siRNA group. Microvascular counting in tumor tissues revealed that MVD in HIF-1α/siRNA group (7.3±1.1) was obviously less than that in SCR/siRNA group (17.2±3.2) (*P*<0.05). (Fig.4)

## DISCUSSION

HIF-1α, up-regulated in most human malignant neoplasm due to intra-tumoral hypoxia, can directly reprogram the metabolic state in cells, and this response is important in hypoxic settings such as vascular disease and cancer.^13^ Various studies have demonstrated that osteosarcoma aberrant blood supply cannot meet the increased demands of oxygen placed on the rapidly expanding tumor when it developed beyond certain region.^1^ So the intra-tumoral microenvironment is always in hypoxic condition associated with the deviant biological and pathological behavior, such as, metabolism, angiogenesis, growth, metastasis, radiotherapy chemotherapy resistance, etc.^3^-^5^,^14^ Hypoxic adaptations are attributed to increasing glucose transportations, glycolysis, especially, angiogenesis. The formation of new blood vessels necessitates VEGF, fibroblast growth factor, angiogenin, etcetera, which are modulated by HIF-1.^9^-^11^ HIF-1 is thought to be critical mediator of the transcriptional response to hypoxia and the expression of HIF-1α, the main activity unit, is regulated by oxygen availability.^3^,^14^

RNAi can silence specific gene expression via the mechanism involved in posttranscriptional gene down-regulation in which double-stranded RNA triggers the destruction of cognate RNAs.^6^,^15^ This is mediated by small RNA comprised of 21-23 nucleotides length termed siRNA, specifically directing cleavage of complementary mRNA targets in a procedure that is commonly considered to be an antiviral cellular defense mechanism.^16^,^17^ RNAi is a widespread silencing mechanism that acts at the transcriptional levels by a RNAi effector complex called RNA-induced initiation of transcriptional gene silencing.^18^ RNAi gene silencing at the posttranscriptional and transcriptional levels can be applied to gene therapy. Several studies managed *in vitro* and *in vivo* documented that RNAi-based remedy can be used for rectifying single-gene or multiple genes disorders with over-expression of relevant proteins.^19^ Blocking the platelet-derived growth factor receptor-b signaling by RNAi prevented pancreatic cancer cells invasion *in vitro* and metastasis *in vivo*.^20^ Some clinical trials had been conducted. Mark *et al* had conducted the first in-human phase I clinical trial involving the systemic administration of siRNA to patients with melanoma, and demonstrated that siRNA administered systemically to a human can produce a specific gene silencing via an RNAi mechanism.^21^

Angiogenesis is a critical process during embryonic development, homeostasis, and tumor initiation and progression.^22^ Albeit beneficial effects on many physiological processes, such as tissue growth and regeneration, angiogenesis also promotes and sustains the pathological growth and metastasis of tumors.^8^ Under the hypoxic tumor microenvironment, tumor tissue produce growth factors termed proangiogenic factor, including VEGF that stimulates angiogenesis via binding VEGF receptor-2.^9^,^10^,^23^ The tumor-promoting effect of VEGF is accompanied by a strong increase in MVD; blocking VEGF and/or its receptor VEGFR-2 inhibits angiogenesis, decreases MVD, impairs tumor renewal capability, and induces tumor regression.^24^ VEGF over-expression induces the proliferation and expansion of CD34+ cancer stem cells.^24^ Generally considered, CD34, a marker of vascular endothelial progenitor cell, is propitious to endothelial repair and regeneration and tumor angiogenesis.^25^ CD34+ cells are dominantly located in vascular endothelium and its immunohistochemistry is normally utilized as a tool for the quantification of MVD.

In the *in vivo* and *in vitro* experimentations, our study demonstrates HIF-1α gene silenced by pSilencer2.1-HIF-1α and corresponding changes of angiogenesis in osteosarcoma. *In vitro*, The results of western blot and qPCR illustrate that siRNA-mediated HIF-1α gene silencing can significantly reduce VEGF protein contents and the expression of VEGF mRNA at transcription level. VEGF immunohistochemistry also indicates this phenomenon. *In vivo*, according to morphological observation, angiogenesis is inhibited in the condition of siRNA-mediated HIF-1α gene silencing. The expressions of VEGF and CD34 are compressed in HIF-1α/siRNA group compared to SCR/siRNA group. MVDs in HIF-1α/siRNA group obviously exhibit decreased angiogenesis. Collectively, blocking HIF-1α transcription activation signal pathway inhibits angiogenesis.

## CONCLUSION

In summary, the siRNA-mediated HIF-1α gene silencing in osteosarcoma have successfully amputated the tumor angiogenesis. Then inhibiting angiogenesis via blocking HIF-1α expression may one of the targeting crucial steps in treating hypoxic tumor cells, is expected to provide a novel and attractive target of therapeutic strategies of osteosarcoma.
